# Solid-state fermentation of distilled dried grain with solubles with probiotics for degrading lignocellulose and upgrading nutrient utilization

**DOI:** 10.1186/s13568-018-0715-z

**Published:** 2018-11-26

**Authors:** Cheng Wang, Weifa Su, Yu Zhang, Lihong Hao, Fengqin Wang, Zeqing Lu, Jian Zhao, Xuelian Liu, Yizhen Wang

**Affiliations:** 1 National Engineering Laboratory of Biological Feed Safety and Pollution Prevention and Control, Key Laboratory of Animal Nutrition and Feed, Ministry of Agriculture, Key Laboratory of Animal Nutrition and Feed Science of Zhejiang Province, Institute of Feed Science, Zhejiang University, 866 Yuhang Tang Road, Hangzhou, 310058 Zhejiang People’s Republic of China; 2 Ningbo Academy of Agricultural Science, 19 Houde Road, Ningbo, 315000 China; 3 Dabeinong Technology Group Co., Ltd., Beijing, China

**Keywords:** DDGS, Digestibility, Nutritional quality, Solid-state fermentation

## Abstract

Solid-state fermentation (SSF) was carried out in this study to improve the nutritional digestibility of two types of distilled dried grain with solubles (DDGS) by inoculating probiotic combinations. The fermented DDGS (FDDGS) contained more crude protein, small peptides and total amino acids than did unfermented DDGS. The concentrations of fiber indexes significantly declined after fermentation. The amounts of probiotics, enzymes and organic acids were significantly improved after fermentation. Microscopy revealed that SSF disrupted the surface structure and increased small fragments of DDGS substrate, thereby facilitating in vitro digestibility of FDDGS. Sodium dodecyl sulfate–polyacrylamide gel electrophoresis and high-performance liquid chromatography indicated the breakdown of macromolecular protein and lignocellulose, which contributed to the increase of small peptides and monosaccharides. These findings suggested the great potential of SSF to promote the nutritional quality and digestibility of the two DDGS and to expand their utilization.

**Electronic supplementary material:**

The online version of this article (10.1186/s13568-018-0715-z) contains supplementary material, which is available to authorized users.

## Introduction

Distilled dried grains with solubles (DDGS), as a by-product from ethanol and alcoholic beverage production, contains high concentrations of crude protein (CP), amino acids (AA), fat, minerals and yeast component (Stein and Shurson 2009). Distilled dried grains with solubles (DDGS) is widely used in animal diets because of its availability and cost-effective benefits for sustainable animal production (Spiehs et al. 2002). The bioethanol industry is well established in the US (Lennartsson et al. 2014). In 2015, the United States produced approximately 40.23 million tons of corn dried distilled grains (CDDGS) (USDA ERS Database 2015). In P. R. China, rice dried distilled grains (RDDGS) is one of native main residues of Chinese yellow wine production. Chinese yellow wine factories produce approximately 4 million tons of RDDGS every year (calculated based on the yearly production of Chinese yellow wine). Huge production and low price of these two types of DDGS indicates their potential value. However, the high content of fiber and low AA digestibility limit the use of DDGS in monogastric animal diets, thereby putting restraints on diversifying the market for DDGS and decreasing bioethanol and wine industry profits.

Lignocellulose feedstock could be utilized with the supplementation of non-starch polysaccharide (NSP) enzymes to improve its nutrition utilization (Zijlstra et al. 2010). Many efforts have been made on adding single carbohydrases or multi-carbohydrases to DDGS-included non-ruminant diets to improve their nutrient digestibility, growth performance and carcass quality, however, the results were variable (Widyaratne et al. 2009; Yanez et al. 2011). Resistant starch (RS) escapes from the industrial production process of DDGS and is poorly digestible (Li et al. 2014). Fiber and RS interact with protein and other grain compounds during the processes of DDGS production to form enzyme-resistant aggregates, which limits interaction between fiber-degrading enzymes and substrates, thereby further hampering the efficiency of exogenous enzymes (Jha et al. 2015). The limited effects of supplemental carbohydrase on DDGS in vivo might also be attributed to reduced activity of enzymes by the complex gastrointestinal environment. Therefore, an effective and efficient bioprocessing technology is urgently needed to develop strategies for addressing this problem.

The use of solid-state fermentation (SSF) in feedstock processing has gained wide attention due to its great ability to improve nutritional value and increase nutrient bioavailability (Canibe and Jensen 2007; Wang et al. 2018). However, few studies about solid-state fermented DDGS were reported. Whether probiotic fermentation could be effective in decamping fiber-starch-protein aggregates in DDGS and in upgrading protein digestibility needs to be further investigated.

We hypothesized that SFF can promote DDGS nutritional value. The objectives of the present study were to investigate: (1) the effects of treating CDDGS and RDDGS with SSF on the change of nutrition composition, microbial metabolites and in vitro digestibility; (2) the microstructure of two types of DDGS following fermentation using scanning electron microscopy (SEM) and confocal laser scanning microscopy (CLSM); and (3) the change of protein and lignocellulosic profiles by sodium dodecyl sulfate–polyacrylamide gel electrophoresis (SDS-PAGE) and high-performance liquid chromatography (HPLC).

## Materials and methods

### Microorganisms, basal substrate and solid-state fermentation

Two commercial DDGS samples (1 CDDGS and 1 RDDGS) were used. The CDDGS was of American origin and was obtained from a local feed mill (Zhejiang Kesheng feed Co., LTD, Zhejiang, China). The RDDGS was from Zhejiang Gu Yue Long Shan Shaoxing Wine Co., Ltd.

*Bacillus subtilis* CW4 and *Lactobacillus plantarum* CWLP were obtained from traditional Chinese pickled vegetables. Both microorganisms were confirmed with 16S rDNA sequencing and deposited in CGMCC. The CGMCC number and 16S rRNA gene of CW4 and CWLP are listed in supplementary materials (Additional file 1: Tables S1, S2). The NCBI accession numbers of CW4 and CWLP are MH885533 and MH885501 respectively. For inoculum preparation, microorganisms were first activated by culture in Luria broth (LB) liquid medium for *B. subtilis* and de Man, Rogosa and Sharp (MRS) liquid medium for *L. plantarum* at 37 °C and 150 rpm for 12 h. The activated culture (population of 10^7−8^ CFU/mL) was used as the inoculum for the fermentation of DDGS.

The 20 g basal substrates (80% DDGS and 20% wheat brane) were mixed and inoculated with 6% (v/w) (1.0 × 10^8^ CFU/mL) of *B. subtilis CW4*. The substrate was fermented in a 100 mL Erlenmeyer flask and covered with a sterile membrane (aerobic condition), and sterile water was added to achieve a 48% moisture content in dry basis. The substrate was fermented at 37 °C for 36 h. After the first-stage fermentation, the fermented mixture was inoculated with 3% (v/w) (1.0 × 10^8^ CFU/mL) of *L. plantarum,* covered with a rubber plug (anaerobic condition) and incubated in an anaerobic condition at 37 °C (the second-stage of fermentation). After 48 h of anaerobic fermentation, some of the wet samples were collected for immediate testing for microbial metabolites and in vitro digestibility. The rest of the samples were treated at 105 °C for 20 min to stop fermentation. Then, samples were dried at 60 °C for 36 h, cooled and ground. Dry and wet samples were used for nutritional analysis.

### Chemical analyses

Dried samples were ground and sieved through a 1-mm screen and then were analyzed for dry matter (DM), CP, AA, ether extract (EE), neutral detergent fiber (NDF), acid detergent fiber (ADF), ash, calcium (Ca), and total phosphorus P as described by the AOAC (2005). Trichloroacetic acid–soluble protein (TCA-SP) was analyzed as reported by Ovissipour et al. (2009). The chemical results of the DDGS before and after fermentation are presented in Table 1.

**Table 1:** Nutrient composition of two DDGS before and after fermentation (as air-dry basis)

Item, %	CDDGS		RDDGS	
	Unfermented	Fermented	Unfermented	Fermented
Dry matter	89.31 ± 1.56	91.10 ± 1.93	88.06 ± 1.03	89.21 ± 1.78
Lignin	1.77 ± 0.10^a^	1.10 ± 0.12^b^	1.75 ± 1.56^a^	1.28 ± 1.60^b^
Cellulose	5.70 ± 0.62^a^	3.55 ± 0.51^b^	5.30 ± 0.50^a^	3.42 ± 0.27^b^
ADF	10.35 ± 1.68^a^	4.94 ± 0.43^b^	11.86 ± 1.38	8.78 ± 1.50
NDF	30.24 ± 2.62^a^	20.35 ± 2.92^b^	20.19 ± 1.62^a^	16.27 ± 0.85^b^
Total dietary fiber	12.15 ± 0.36	12.98 ± 0.60	11.4 ± 41.37	13.35 ± 0.52
Total starch	14.28 ± 0.95	12.99 ± 2.06	16.14 ± 2.36^a^	11.94 ± 0.65^b^
Amylopectin	9.62 ± 1.06	9.75 ± 1.92	11.87 ± 1.62^a^	9.00 ± 0.46^b^
Amylose	4.66 ± 0.52^a^	3.24 ± 0.33^b^	4.27 ± 0.77^a^	2.95 ± 0.20^b^
Crude protein	27.23 ± 0.62^b^	31.38 ± 0.55^a^	34.05 ± 0.46^b^	37.51 ± 0.47
TCA-SP	12.47 ± 0.44^b^	15.57 ± 0.64^a^	17.32 ± 0.64^b^	22.61 ± 0.98^a^
FAA	0.25 ± 0.04^b^	0.53 ± 0.05^a^	0.74 ± 0.06^b^	3.20 ± 0.35^a^
Small peptides	12.22 ± 0.46^b^	15.04 ± 0.60^a^	16.58 ± 0.59^b^	19.42 ± 0.63^a^
EE	5.88 ± 0.20	6.19 ± 0.28	3.06 ± 0.09	3.46 ± 0.17
Ash	4.22 ± 0.65	3.28 ± 0.43	4.81 ± 0.32	3.83 ± 0.34
Ca	0.14 ± 0.02	0.16 ± 0.01	0.22 ± 0.02	0.18 ± 0.02
P	0.31 ± 0.03	0.28 ± 0.01	0.29 ± 0.02^a^	0.17 ± 0.05^b^
Indispensable AA				
Arg	1.33 ± 0.09	1.41 ± 0.06	1.12 ± 0.19	1.16 ± 0.18
His	0.43 ± 0.02^b^	0.72 ± 0.03^a^	0.50 ± 0.02	0.48 ± 0.01
Ile	1.21 ± 0.03	1.25 ± 0.14	1.20 ± 0.02	1.20 ± 0.06
Leu	2.03 ± 0.08	2.14 ± 0.14	1.56 ± 0.07^b^	2.30 ± 0.17^a^
Lys	0.47 ± 0.01^b^	0.56 ± 0.02^a^	0.40 ± 0.02^b^	0.56 ± 0.02^a^
Met	0.49 ± 0.01^b^	0.53 ± 0.01^a^	0.36 ± 0.04^b^	0.65 ± 0.05^a^
Phe	1.14 ± 0.12^b^	1.47 ± 0.13^a^	1.13 ± 0.17	1.36 ± 0.10
Thr	0.91 ± 0.02	1.05 ± 0.16	0.58 ± 0.04^b^	1.03 ± 0.19^a^
Val	1.30 ± 0.06^b^	1.52 ± 0.04^a^	1.13 ± 0.09^b^	1.30 ± 0.05^a^
Dispensable AA				
Asp	1.81 ± 0.05^b^	2.23 ± 0.09^a^	1.25 ± 0.06^b^	1.44 ± 0.05^a^
Ser	1.21 ± 0.02	1.22 ± 0.03	1.16 ± 0.16^b^	1.81 ± 0.10^a^
Glu	5.00 ± 0.19^b^	5.42 ± 0.11^a^	3.87 ± 0.12^b^	4.26 ± 0.05^a^
Gly	1.10 ± 0.01	1.16 ± 0.06	1.03 ± 0.06	1.06 ± 0.05
Ala	1.32 ± 0.06^b^	1.58 ± 0.10^a^	1.34 ± 0.11^b^	2.02 ± 0.18^a^
Cys	0.39 ± 0.03	0.47 ± 0.09	0.70 ± 0.03	0.77 ± 0.05
Tyr	1.25 ± 0.05	1.32 ± 0.04	1.79 ± 0.17	1.52 ± 0.15
Pro	1.16 ± 0.17	1.23 ± 0.04	1.14 ± 0.04	1.20 ± 0.01
Total AA	22.55 ± 0.46^b^	25.3 ± 10.74^a^	20.29 ± 0.37^b^	24.13 ± 0.84^a^

Values are means of three replicates per treatment. Means in a row without common superscript differ significantly (*P *< 0.05)

### Microorganisms and microbial metabolites

The pH and microbiological counts were analyzed as described by (Wang et al. 2017). Briefly, A 2-g wet sample was dissolved in 20 mL of water and centrifuged at 8000 r min^−1^ for 10 min. A pH meter (METTLER TOLEDO, Switzerland) was used to determine the pH of the supernatant.

Ten-fold dilutions of the feed samples were prepared for microbial enumeration. *B. subtilis* was counted on LB agar following aerobic incubation at 37 °C for 48 h. *L. plantarum* were counted on MRS agar at 37 °C for 48 h. The numbers of *Enterobacteria* were measured on McConkey agar following aerobic incubation at 37 °C for 24 h. The mold was counted on Salt Czapek Dox agar following aerobic culturing at 28 °C for 96 h.

Organic acids were determined by HPLC as described by (Khajeh et al. 2015)

The activity of β-mannanase, xylanase and cellulase was analyzed by the DNS method described by (Wongputtisin et al. 2012). One unit (U) of enzyme was defined as the amount of enzyme that produces 1 µmol of reducing sugar in 1 min. The activity of neutral protease, acid protease and alkaline protease was determined as follows using a slightly modified method reported by (Ueda et al. 2007). A unit of protease activity was defined as the amount of enzyme that generates 1 μg of tyrosine per minute per mL of reaction mixture.

### In vitro digestibility

The in vitro digestibility values of the DDGS samples were analyzed at the Institute of Animal Sciences, Chinese Academy of Agricultural Sciences as slightly modified by (Zhao et al. 2014). A computer-controlled, simulated digestion system was applied to accurately predict the digestibility of monogastric animals. Briefly, simulated gastric fluid was composed of approximately 1550 U/mL pepsin (Sigma 10070; Sigma-Aldrich Co., St. Louis, MO). The small intestinal fluid was simulated with 4730 U/mL of amylase (Sigma A3306; Sigma-Aldrich Co.), 550 U/mL of trypsin (Amresco 0785; Amresco Inc., Solon, OH), and 154 U/mL of chymotrypsin (Amresco 0164; Amresco Inc.). Before in vitro intestinal digestion, 2 mL of small intestinal fluid was added to a digestion chamber. The small intestinal fluid was diluted by 20 mL residual simulated gastric fluid, which reached a neutral pH after 3 washing procedures during in vitro gastric digestion. The residues were centrifuged at 3000×g for 15 min, and the sediments were dried at 105 °C for 5 h and tested in subsequent DM, CP, gross energy (GE) and AA contents.

### Microscopic analyses

For SEM, the microstructure of uninoculated DDGS and FDDGS was observed using a field-emission scanning electron microscope (KYKY-EM3200, Shanghai, China) at 100×, 700×, and 3000× magnification. The freeze–dried samples were placed on an aluminum stub and coated with gold. The micrographs were taken at 25 kV and a high vacuum mode.

For CLSM, proteins were labeled with fluorescein isothiocyanate (FITC), lignocellulose was labeled with calcofluor white (CW), and starch was labeled with Concanavalin A (Con A) (Wang et al. 2012). In total, 10 g/L of the FITC solution, 250 mg/L of the Con A solution and 300 mg/L of the CW solution were added to the samples successively and incubated for 1 h, 30 min and 30 min. The mixtures were washed with distilled, deionized water twice after each staining. Stained samples were placed on glass slides and observed under a confocal laser scanning microscope (LSM 710; Carl Zeiss MicroImaging GmbH, Jena, Germany). The excitations were at 518 nm, 440 nm and 668 nm for FTTC, CW and Con A, respectively. Images of the microstructures of DDGS were recorded with ZEN 2010 software (Carl Zeiss MicroImaging GmbH). Protein, fiber and starch turn green, blue and red, respectively, at the three different excitations. The fluorescence intensity indicates the content of each nutrient.

### Sodium dodecyl sulfate–polyacrylamide gel electrophoresis (SDS-PAGE)

Proteins before and after fermentation of the two DDGS were extracted as described by (Hamaker et al. 1995). Both prolamins and nonprolamins of each sample were pooled together. The concentration of protein in the samples was determined with the BCA protein assay kit (keyGEN bioTECH, Shanghai, China). Protein samples were fractionated by an SDS-PAGE system based on 12% polyacrylamide separating gels containing 0.1% SDS in Tris–glycine buffer. Approximately 6 µg of protein sample was added to each well, followed by separation at 60 mV for 210 min. The gel was stained with Coomassie Brilliant Blue (CBB) R-250 (Bio-Rad, California, USA) for 60 min and de-stained with 8% acetic acid.

### High-performance liquid chromatography (HPLC)

Monosaccharide analysis was conducted with an HPLC with a vacuum degasser, binary pump, column heater, and diode-array detection system. The column used was a 250 mm × 4.5 mm i.d., 5 μm particle size, C18XBridge from Waters (Biotech. Company, Dublin, Ireland) with a C18 security guard column.

The 200 μL samples were derivatized with 50 µL of 0.5 mol/L 1-phenyl-3-methyl-5-pyrazolone (PMP) and 50 µL of 0.3 mol/L in an alkaline environment in a 70 °C-water bath for 60 min. Then, samples were cooled and extracted by trichloroethane for 10 min.

Analysis was performed at 40 °C. The mobile phase was prepared from potassium phosphate monobasic aqua (50 mM, pH = 5.5) and acetonitrile as eluent A and B (78:22 v/v). The flow-rate was 1 mL·min^−1^. The injection volume was 10 μL. Monitoring was performed at 245 nm.

### Analytical methods

Computer-controlled simulated nutrient digestibility (%) = (original nutrient amount − residual nutrient amount)/original nutrient amount × 100%.

All data were analyzed using SPSS software (SAS Inc., Chicago, IL). One-way ANOVA analysis followed by Tukey’s multiple comparison test was used to determine the statistical significance of multiple comparisons in the results of HPLC, and independent sample *t*-tests were used for comparisons of chemical composition, microbial metabolites and digestibility before and after fermentation. The differences between the treatments’ means were considered significant at *P *< 0.05 and were considered trends at *P* < 0.10.

## Results

### Chemical composition

The analyzed nutrient contents of the two types of DDGS before and after fermentation are presented in Table 1. The contents of NDF and insoluble non-starch polysaccharides (NSP) in CDDGS were 30.24 ± 2.62 and 17.32 ± 1.69, respectively, which was approximately 1.5 times higher than that of RDDGS. Remarkably, RGGDS contained more CP, TCA-SP, free amino acids (FAA) and small peptides compared to CDDGS.

Compared with unfermented DDGS, the inoculated DDGS contained less CF, insoluble NSP and amylose, which were declined (*P *< 0.05) by approximately 36, 28%, and 30%, respectively, after the treatment. Similarly, in CDDGS, the content of lignin, ADF and NDF decreased to 1.10 ± 0.12, 4.94 ± 0.43 and 20.35 ± 2.92, respectively, while that of RDDGS reduced by 26.86%, 25.97% and 19.42%, respectively. Notably, the content of TCA-SP (< 10 kDa), FAA and small peptides in uninoculated CDDGS was 12.47, 0.25 and 12.2%, respectively, which was increased 1.25, 2.12 and 1.23-fold, respectively, in inoculated CDDGS. Similar trends can be observed in RGGDS. The fermentation tended to promote the content of TCA-SP, FAA and small peptides of RDDGS to 22.61, 3.20 and 19.42%, respectively.

Inoculating with probiotics also greatly affected the AA composition patterns of CDDGS and RDDGS (Table 1). In the present study, three indispensable AA (Lys, Met and Val) and three dispensable AA (Asp, Glu and Ala) significantly increased in both FDDGS compared to unfermented DDGS. The concentrations of His and Phe of FCDDGS were improved by 67.44% and 28.95%, respectively, compared to CDDGS, while RDDGS contained more Leu, Thr and Ser after fermentation. Notably, total AA increased by 12.20% and 18.93% individually in the two DDGS.

### Microbial metabolites

To further investigate the nutritional value of FDDGS, we determined microbial metabolites after fermentation. The analyzed microbial metabolites of the DDGS and FDDGS are presented in Table 2.

**Table 2:** Microbial metabolites of two DDGS before and after fermentation (as-fed basis)

Item, %	CDDGS		RDDGS	
	Unfermented	Fermented	Unfermented	Fermented
Microorganism				
*Lactobacillus plantarum*, × 10^8^ CFU/g	ND	6.70	ND	5.30
*Bacillus subtilis*, × 10^8^ CFU/g	ND	5.00	ND	5.20
*Enterobacterium*, × 10^4^ CFU/g	3.63	ND	2.68	ND
Mold, × 10^2^ CFU/g	3.10	ND	3.60	ND
Total probiotics, × 10^8^ CFU/g		11.70		10.50
Organic acids, mmol/Kg				
Lactic acid	21.48 ± 3.77^b^	232.10 ± 20.3^a^	14.30 ± 1.89^b^	115.21 ± 12.1^a^
Formic acid	9.35 ± 0.79	8.68 ± 0.46	14.43 ± 1.14^b^	22.65 ± 2.97^a^
Acetic acid	1.59 ± 0.30^b^	16.15 ± 2.07^a^	17.35 ± 1.63^b^	25.27 ± 4.24^a^
Propionic acid	0.03 ± 0.00^b^	0.83 ± 0.11^a^	0.03 ± 0.00^b^	0.40 ± 0.04^a^
Butyric acid	33.04 ± 0.53	32.90 ± 0.39	9.41 ± 0.46	10.94 ± 1.40
pH	6.39 ± 0.04^a^	4.21 ± 0.08^b^	6.43 ± 0.10^a^	4.34 ± 0.16^b^

Values are means of three replicates per treatment. Means in a row without common superscript differ significantly (*P* < 0.05)

Notably, all probiotics proliferated to 10^8^ CFU/g, whereas pathogens such as enterobacterium and molds declined to undetected level after fermentation in the present study. The number of total probiotics reached 11.70 × 10^8^ CFU/g and 10.50 × 10^8^ CFU/g in FCDDGS and FRDDGS, respectively.

**Fig. 1:**
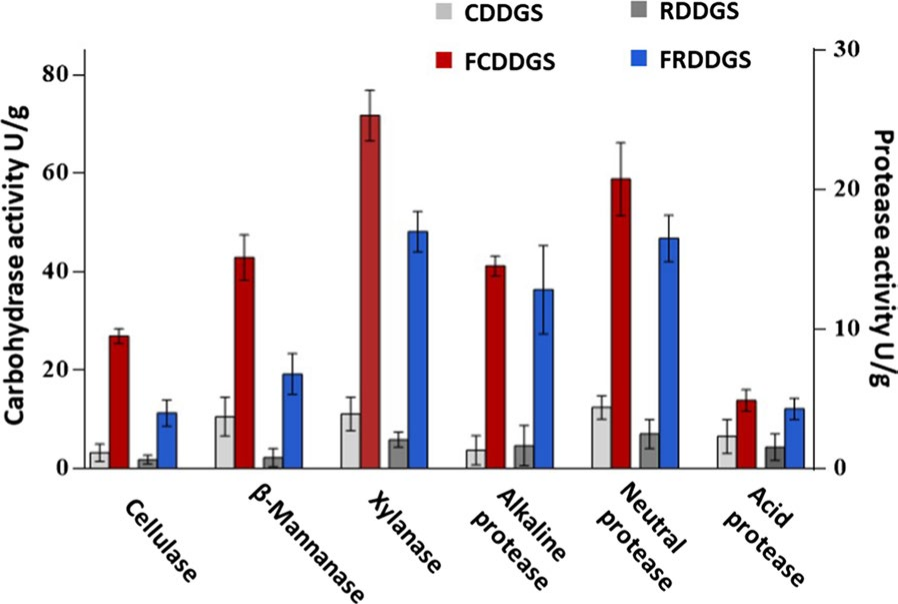
Carbohydrase and protease activities of two DDGS before and after SSF. The activity of carbohydrase and protease are shown on the left and right longitudinal axis, respectively

Carbohydrase and proteases were determined in DDGS and FDDGS (Fig. 1). All the determined enzymes were significantly improved after inoculation. Remarkably, xylanase and neutral protease were the most secreted enzymes detected after fermentation.

In addition, *Lactobacillus* spp. can efficiently produce lactic acid and decrease the pH of the substrates to prevent pathogens and promote palatability (Missotten et al. 2015). Therefore, four kinds of organic acids were also tested (Table 2). The concentration of lactic acid increased by approximately 10 and 8 times in FCDDGS and FRDDGS, respectively, compared to that in unfermented substrates. Similarly, the content of acetic acid and propionic acid was substantially improved by inoculating the probiotics. The pH value dropped to 4.21 ± 0.08 and 4.34 ± 0.16 in FCDDGS and FRDDGS, respectively. In the present study, the concentrations of lactic acid and acetic acid, which significantly reached 150 and 15 mMol/kg, respectively, improved after fermentation. The content of formic acid of FRDDGS increased to 22.65 mMol/kg. Hence, FDDGS could prevent the growth of *Enterobacteriacea, Escherichia coli*, *Salmonella typhimurium* and some molds. The pH values of FCDDGS and FRDDGS were 4.21 and 4.34, respectively.

### In vitro digestibility

The results of digestibility of unfermented and fermented DDGS are presented in Table 3. In vitro DM, CP and gross energy digestibility of inoculated CDDGS were improved (*P *< 0.05) by 7.79%, 11.29% and 11.71%, respectively, compared with uninoculated CDDGS. Similar results were observed in RDDGS. The digestibility of DM, CP and GE significantly increased to 68.84, 78.72 and 73.37%, respectively. In addition, the in vitro digestibility of 9 amino acids, including 6 essential amino acids (Ile, Leu, Lys, Phe Thr and Val) and 3 nonessential AA, improved greatly (*P* < 0.05). Furthermore, the digestibility of average indispensable AA, average dispensable AA and total AA were greatly improved approximately 1.20, 1.13 and 1.18 times, respectively, compared to unfermented DDGS in both types of DDGS. Interestingly, the digestibility of total AA of FRDDGS was 60.54 ± 3.07, which was 1.14 times higher than that of FCDDGS.

**Table 3:** Computer-controlled simulated digestibility of two DDGS before and after fermentation (dry matter basis)

Item, %	CDDGS		RDDGS	
	Unfermented	Fermented	Unfermented	Fermented
Dry matter	48.17 ± 2.05^b^	52.24 ± 1.46^a^	64.48 ± 2.02	68.84 ± 2.80
Crude protein	66.80 ± 2.51^b^	74.34 ± 3.36^a^	70.05 ± 1.29^b^	78.72 ± 1.89^a^
Gross energy	54.04 ± 1.06^b^	60.37 ± 1.72^a^	66.73 ± 1.98^b^	73.37 ± 1.49^a^
Indispensable AA				
Arg	55.02 ± 2.65^b^	66.43 ± 3.63^a^	50.38 ± 3.78	62.39 ± 1.49
His	61.12 ± 2.04	51.66 ± 2.01	57.51 ± 1.96	57.72 ± 1.60
Ile	48.13 ± 2.60^b^	62.00 ± 3.07^a^	48.78 ± 3.53^b^	64.52 ± 4.04^a^
Leu	39.52 ± 2.54^b^	48.78 ± 1.93^a^	43.68 ± 3.10^b^	56.00 ± 2.36^a^
Lys	44.78 ± 2.19^b^	66.34 ± 2.05^a^	57.35 ± 3.81^b^	70.27 ± 2.49^a^
Met	60.52 ± 2.43	60.12 ± 1.40	56.36 ± 1.33^b^	60.36 ± 2.07^a^
Phe	42.36 ± 1.27^b^	53.88 ± 1.06^a^	39.43 ± 3.70^b^	60.14 ± 2.08^a^
Thr	46.59 ± 1.62^b^	57.46 ± 1.46^a^	48.78 ± 3.56^b^	63.53 ± 4.00^a^
Val	50.00 ± 2.42^b^	57.98 ± 1.40^a^	47.27 ± 3.97^b^	59.16 ± 3.10^a^
Average	49.71 ± 1.26^b^	58.81 ± 0.44^a^	49.95 ± 0.81^b^	61.57 ± 0.67^a^
Dispensable AA				
Asp	38.69 ± 3.95^b^	48.65 ± 4.01^a^	49.06 ± 3.13^b^	61.11 ± 4.99^a^
Ser	49.49 ± 2.60^b^	57.51 ± 1.70^a^	47.12 ± 3.12^b^	58.70 ± 1.98^a^
Glu	46.90 ± 1.61	51.26 ± 2.33	49.25 ± 3.08	55.43 ± 2.76
Gly	43.00 ± 2.04^b^	52.87 ± 3.20^a^	46.20 ± 1.56	47.05 ± 1.57
Ala	44.85 ± 1.24^b^	54.12 ± 1.62^a^	54.96 ± 2.40^b^	64.80 ± 2.10^a^
Cys	53.44 ± 4.34	46.67 ± 3.03	48.83 ± 3.56	43.36 ± 2.42
Tyr	56.81 ± 1.46	59.70 ± 2.58	27.60 ± 2.78^b^	42.27 ± 3.90^a^
Pro	36.91 ± 2.71	41.81 ± 3.57	49.50 ± 2.07	51.31 ± 2.30
Average	46.26 ± 1.37^b^	51.58 ± 1.76^a^	46.56 ± 0.97^b^	52.97 ± 1.62^a^
Total AA	46.68 ± 1.84^b^	53.24 ± 1.40^a^	49.98 ± 3.0^b^	60.54 ± 3.07^a^

Values are means of three replicates per treatment. Means in a row without common superscript differ significantly (*P* < 0.05)

### Microscopic observation

**Fig. 2:**
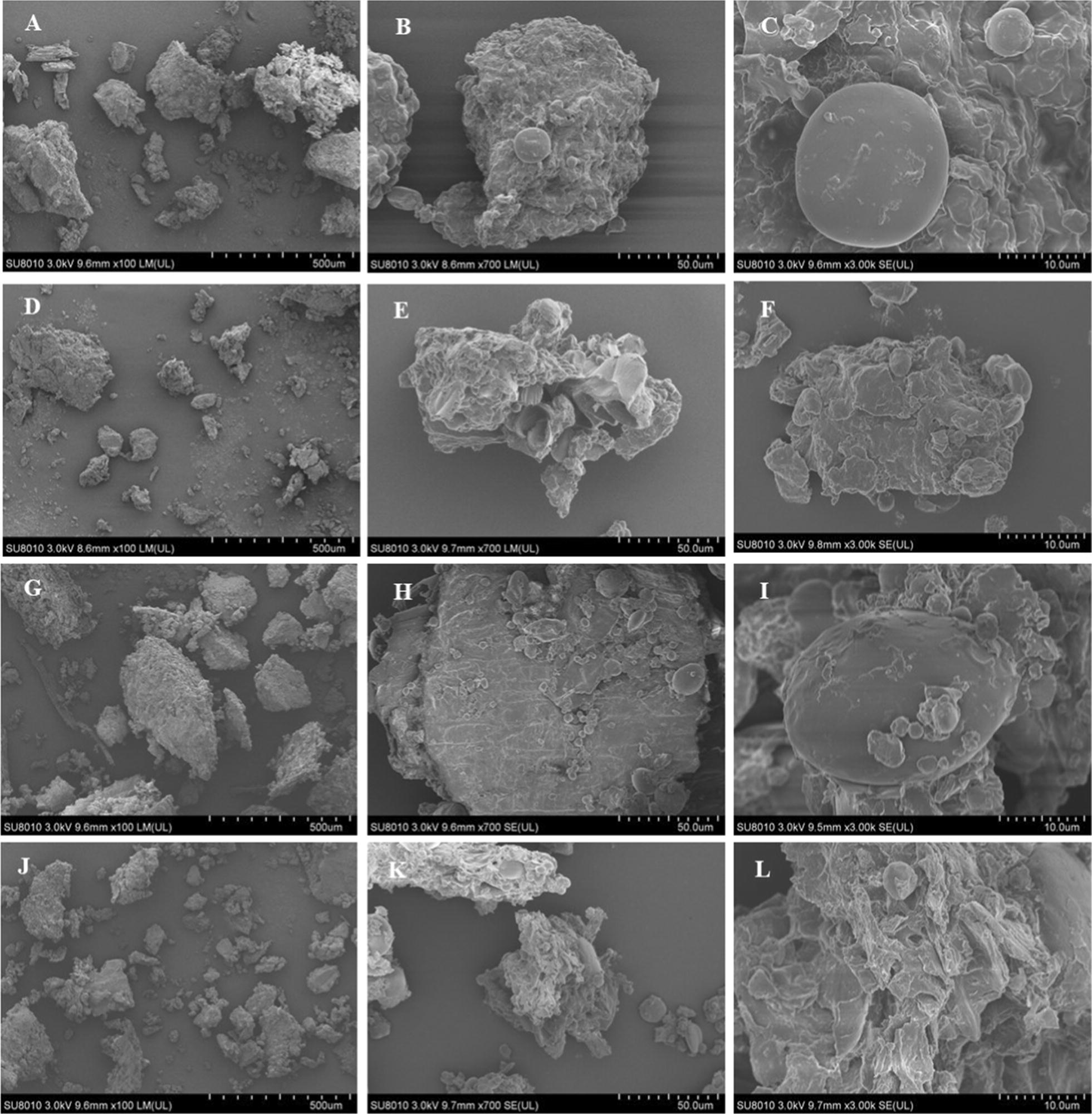
Scanning electron microscope images (×100, ×700, ×3000) of fermented residues of CDDGS (**A**, **B**, and **C**), FCDDGS (**D**, **E**, and **F**), RDDGS (**G**, **H**, and **I**) and FRDDGS (**J**, **K**, and **L**), respectively

The microstructure was clearly different between DDGS and untreated DDGS in both SEM and CLSM. Figure 2 shows the morphological features of CDDGS and RDDGS before and after fermentation by SEM at 100-, 700- and 3000-fold magnifications. At 100-fold magnification, FCDDGS was more fragmentized than CDDGS. More holes were observed in FCDDGS at 700-fold magnification compared to CDDGS, whereas unfermented CDDGS had a relatively smooth surface. At the highest magnification, intact starch granules were found in FDDGS. However, the starch granules in CDDGS were incomplete and cracked. Similarly, FRDDGS contained smaller fragments, large holes and more irregular surfaces than RDDGS.

**Fig. 3:**
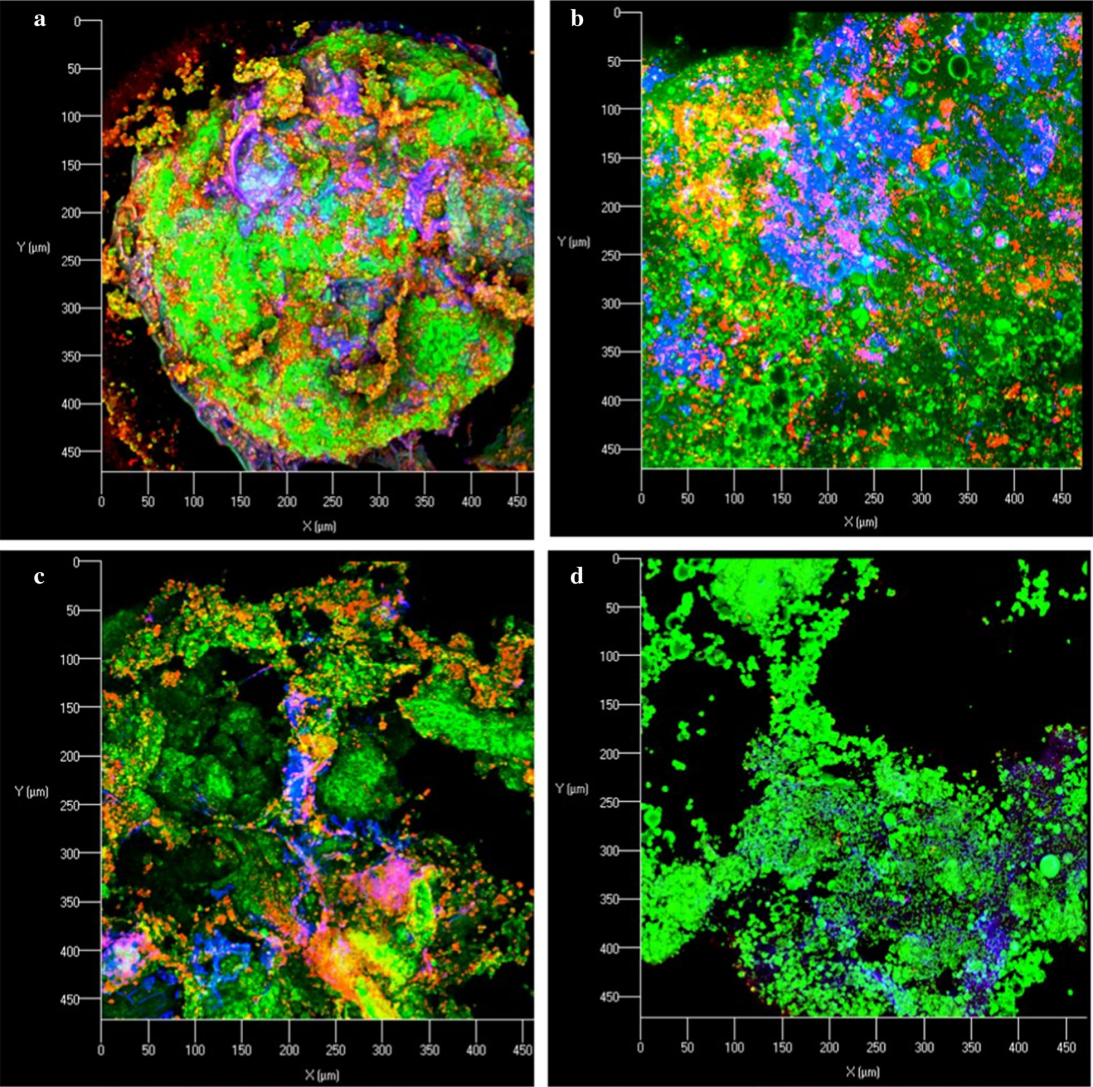
Confocal laser scanning microscope images (475 μm × 475 μm) of fluorescein isothiocyanate (FITC), calcofluor white (CW) and Concanavalin A (Con A)-stained CDDGS (**a**), RDDGS (**b**), FCDDGS (**c**) and FRDDGS (**d**), respectively

In CLSM images, some large particles of fiber can be observed, which show as dense aggregates. Starch residues (from partially degraded starch granules), protein and other fiber residues of untreated DDGS (Fig. 3a, b) formed complexed aggregates. Additionally, CDDGS had more starch residues and fiber than RDDGS. The fluorescence intensity indicated that the content of resistant starch and fiber remarkably decreased after SSF in the two DDGS, whereas that of protein did not change much.

Both SEM (Fig. 2) and CLSM (Fig. 3) images showed that pieces of the residues were smaller for probiotic-treated DDGS samples than for untreated DDGS samples. The fiber-starch-protein complex structure of DDGS was destroyed, and the content of RS and fiber was obviously decreased after SSF.

### Sodium dodecyl sulfate–polyacrylamide gel electrophoresis (SDS-PAGE)

**Fig. 4:**
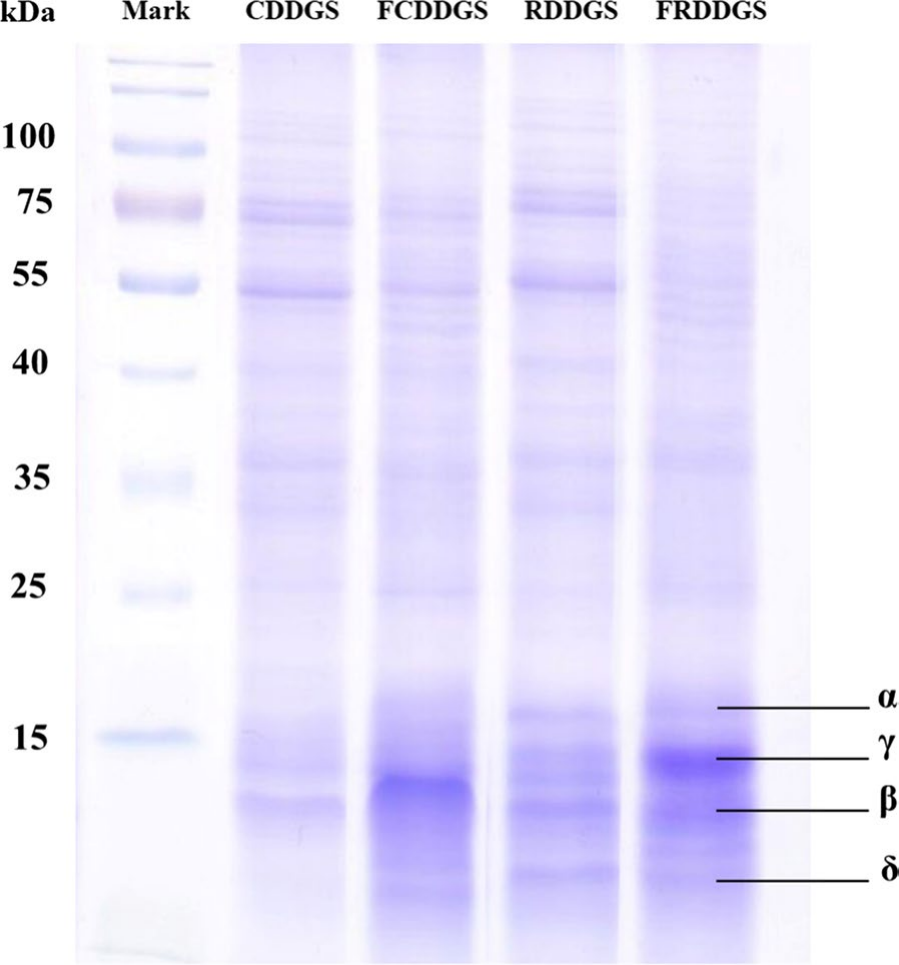
Sodium dodecyl sulfate polyacrylamide gel electrophoresis (SDS-Page) of CDDGS, FCDDGS, RDDGS and FRDDGS

Multiple bands of the protein profile in the range of 13–75 kDa in fermented and unfermented DDGS are shown in Fig. 4. The protein profiles of grains are believed to consist of albumin, globulin, glutelin, and prolamin, among which prolamin constitutes the majority (Guo et al. 2013). DDGS, as a by-product of grains, also contains these four types of protein and some protein residue escape from ethanol and wine production. Subunits of prolamin, including α, β, γ and δ, were separated in DDGS. Proteins of CDDGS and RDDGS formed the typical banding patterns described in previous studies as α, β-, γ- and δ-prolamins protein for CDDGS and RDDGS. SSF significantly affected the characteristics of proteins in DDGS. Similarly, large-sized protein (> 50 kDa) was almost completely removed after fermentation in the two DDGS in the present study. The α and γ subunits of prolamins in the unfermented DDGS were also degraded during SSF. In contrast, fermentation improved the concentration of small peptides (< 15 kDa) in FDDGS compared with that in DDGS.

### Chromatographic analysis

**Fig. 5:**
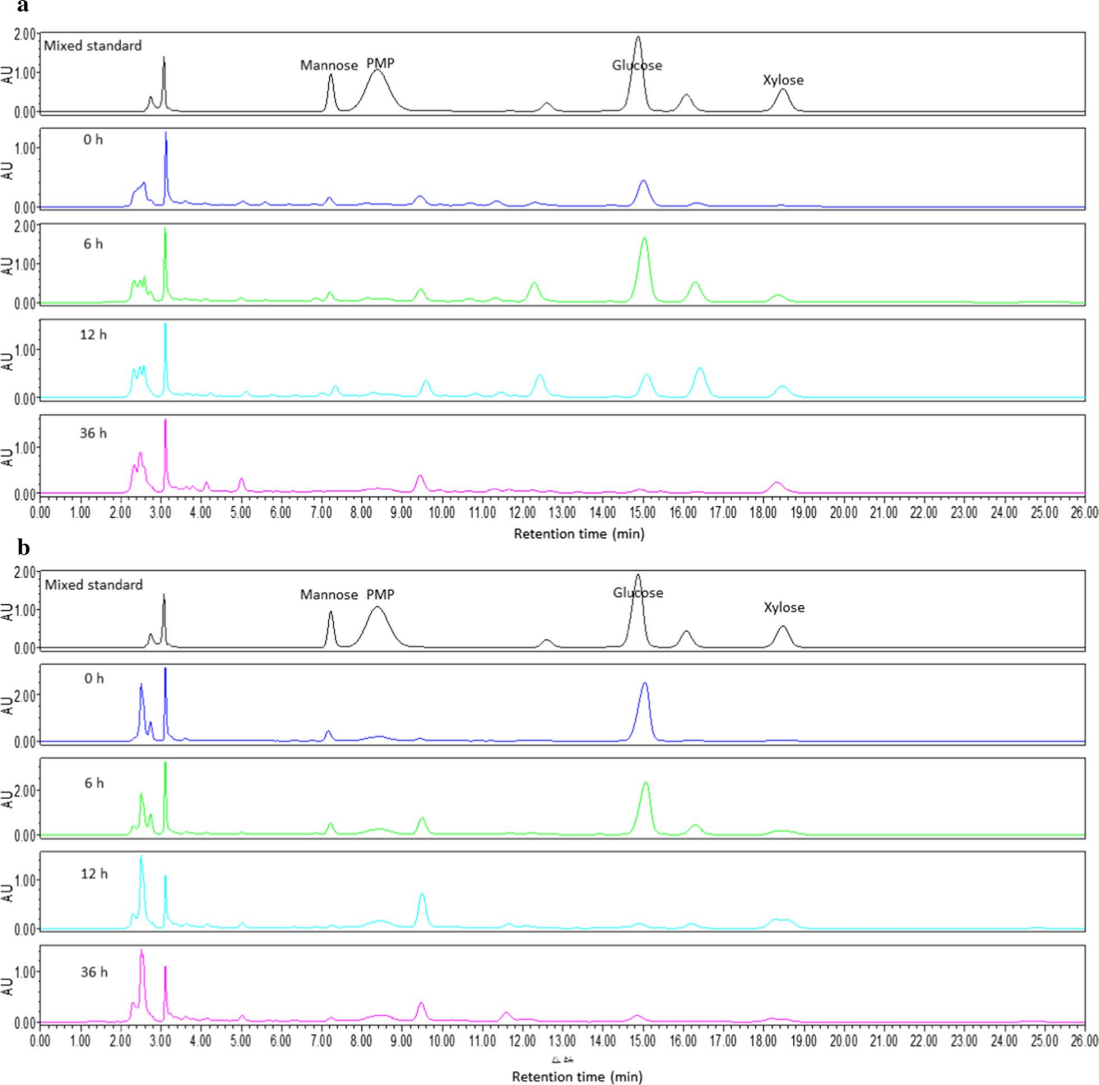
Chromatogram of CDDGS (**a**) and RDDGS (**b**) at 0 h, 6 h, 12 h and 36 h fermentation time, respectively

To further investigate degradation of lignocellulose in DDGS during fermentation, HPLC was employed for the analysis of cellobiose and glucose in the two DDGS at 0 h, 6 h, 12 h and 36 h with pre-column derivatization. The presence of PMP- derivatization monosaccharide peaks of glucose, mannose and xylose was confirmed by standard chromatograms (Fig. 5) and quantitatively analyzed in Table 4. Uninoculated CDDGS contained a small amount of mannose. However, mannose content significantly increased to 0.63 ± 0.04 mmol/kg after 6 h of fermentation followed by a gradual decrease at 12 h and 36 h. The same trends can be found in the concentration of glucose and xylose of FDDGS during the fermentation process. The content of these two monosaccharides reached the peak at 6 h and declined in the next 30 h. In RDDGS, the concentration of mannose and xylose were also greater at 6 h compared to that at 0 h, indicating lignocellulosic hydrolysis. Interestingly, the amount of glucose gradually declined during fermentation. A rate of probiotic utilization higher than the rate of cellulose and hemicellulose degradation may lead to the result.

**Table 4:** Measurement of three monosaccharides in two DDGS at 0 h, 6 h, 12 h and 36 h during fermentation

Item, mmol/Kg	FCDDGS				FRDDGS			
	0 h	6 h	12 h	36 h	0 h	6 h	12 h	36 h
Mannose	0.17 ± 0.01^b^	0.63 ± 0.04^a^	0.23 ± 0.01^b^	0.02 ± 0.00^c^	0.57 ± 0.04^a^	0.63 ± 0.01^a^	0.06 ± 0.01^b^	0.08 ± 0.01^b^
Glucose	1.35 ± 0.04^b^	5.16 ± 0.06^a^	1.38 ± 0.05^b^	0.15 ± 0.01^c^	8.07 ± 0.11^a^	7.20 ± 0.13^b^	0.23 ± 0.02^c^	0.31 ± 0.01^c^
Xylose	0.03 ± 0.00^b^	0.60 ± 0.02^a^	0.72 ± 0.02^a^	0.67 ± 0.02^a^	0.15 ± 0.01^d^	0.79 ± 0.01^b^	0.93 ± 0.02^a^	0.28 ± 0.03^c^

Values are means of three replicates per treatment. Means in a row without common superscript differ significantly (*P *< 0.05)

## Discussion

Consistent with previous reports (Nitrayova et al. 2012; Xue et al. 2012), the nutritional composition was different between the two DDGS. This may be attributed to more complete fermentation of RDDGS than of CDDGS during industrial production. Distinct substrates and formation conditions might also be the results of the difference.

The production of relevant enzymes by *B. subtilis*, such as multi-carbohydrase, amylase and phytase, may cause the breakdown of these lignocellulosic components (Seo and Cho 2016). TCA-SP consists of small-sized peptides and free AA (Gilbert et al. 2008). Animal gastrointestinal tracts can directly absorbed di- and tripeptides (Daniel and Kottra 2004). Additionally, small-sized peptides are assumed to exert antimicrobial activity (Liu et al. 2013). The increased amount of small peptides in FDDGS might be due to the digestion of macromolecular protein in unfermented substrate by proteases secreted by the probiotics (da Silva 2018). Additionally, in both CDDGS and RDDGS, the content of total dietary fiber, CP and EE were increased, which might have been due to the DM (mainly carbohydrates) loss during the fermentation process (Shi et al. 2017). Microbial protein synthesis could also be another reason for the increased protein content (Nguela et al. 2016). Therefore, decreased fiber and increased TCA-SP and small peptide content contributed much to the nutritional value promotion of DDGS. Lys and Met are major limiting amino acids for pigs (NRC 2012). Thus, the amount of Lys and Met is the important consideration in the nutritional quality of feed ingredients. Degradation of macromolecular protein might contribute to the results. Additionally, some microbial AA may be synthesized during bacteria proliferation (Metges 2000). The result was similar to that reported by (Wang et al. 2017), who found that the Lys, Met, Asp and Ala contents were improved in *B. coagulans*-fermented DDGS, suggesting an ideal amino acid pattern for animals.

Probiotics exert their beneficial effects by directly interfering with pathogens by competing with nutrients and adhesion sites (Sanchez et al. 2017) and reinforcing the tight junctions between enterocytes (Ruiz et al. 2016). Abundant probiotics suggest that FDDGS can benefit animals by providing abundant probiotics and by inhibiting pathogens. Moreover, in addition to live probiotics, their metabolites such as digestive enzymes (Kim et al. 2007) and organic acids (Gao et al. 2012) may play important roles in improving the nutritional quality of DDGS here. Increased activities of enzymes after fermentation suggested that the probiotics used can secrete a relatively high-activity and complete enzyme system, which is more effective and efficient than directly adding exogenous enzymes to improve the nutritional composition of feedstock. *B. subtilis* is effective at degrading anti-nutritional factors and large-sized nutrients due to abundant extracellular enzyme secretion (Arevalo-Villena et al. 2017; Chi and Cho 2016). Therefore, the enzymes may come predominantly from first-state aerobic fermentation. Remarkably, xylanase and neutral protease were the most secreted enzymes detected after fermentation, suggesting that these two enzymes may play key roles in changing the nutritional quality of DDGS during the process. Canibe et al. (2001) reported that adding approximately 20 mMol/kg of formic acid into the liquid fermented substrate did not impede the lactic acid bacteria but inhibit the blooming of Enterobacteriaceae. The lactic acid concentration should be above 150 mmol/L to prevent the growth of *E. coli* and molds (van Winsen et al. 2001b). van Winsen et al. (2001a) also showed that supplementing lactic acid and acetic acid to fermented substrate reduced the survival of *S. typhimurium*. The pH values of the final fermented feedstock between 4.0 and 5.0 do not indicate over fermentation or uncontrolled fermentation (Canibe and Jensen 2012; Missotten et al. 2015). The pH values of FCDDGS and FRDDGS suggested proper fermentation of DDGS. Therefore, the content of organic acids increased the biosafety and nutritional quality of FDDGS.

The digestibility of total AA of FRDDGS was higher than that of FCDDGS, which may be due to FRDDGS containing more small-sized peptides than FCDDGS contains. Resistant starch is poorly digested in the upper gut of monogastric animals (Regmi et al. 2011). Instead, pepsin and pancreatin can digest protein better than they can digest fiber (Yang et al. 2010). One of the likely reasons for the higher protein digestibility in animals fed fermented substrates is associated with the reduced gastric pH. Low gastric pH allows more time for digestion in the stomach by reducing the gastric emptying rate and promoting proteolytic activity (Lyberg et al. 2006). FDDGS contained less fiber, more digested protein (small peptides) content and a lower pH value than DDGS. Therefore, the digestibility of DM, protein and some AA of FDDGS was significantly improved in this study. In addition, the increase in digestibility of GE may suggest lipase production and fat predigestion during the process.

This evidence further verified the nutritional composition change of DDGS during the treatment. Previous studies also reported that solid-state fermentation disrupted the surface structure of lignocellulose biomass such as rapeseed meal (Shi et al. 2016; Wang et al. 2012). However, the inoculation used was *Aspergillus* spp., or the fermentation time was long due to composting. In the present study, *Bacillus* spp. disintegrated the complex network of fiber-starch-protein aggregates during the 36-h aerobic fermentation, demonstrating their effective and efficient capability of producing extracellular hydrolases. The results of SEM and CLSM further confirmed that the higher digestibility of FDDGS compared to that of DDGS may be the result of most fiber, starch and protein in FDDGS being separated from each other, thereby being more accessible to digestive enzymes. In contrast, the fiber–starch–protein aggregates of DDGS might be resistant to enzymatic hydrolysis.

Previous studies reported an increase in small peptides and a decrease in macromolecular protein among different fermented substrates after fermentation (Wang et al. 2018). The results of SDS-Page were consistent with the content of TCA-SP and small peptides of DDGS, which were significantly improved after fermentation. This may be attributed to degradation of macromolecular protein in uninoculated substrates. The protein degradation process most likely occurred in the first-state fermentation because the anaerobic fermentation produces substantial organic acids, inhibiting the hydrolytic effects of neutral and alkaline proteases.

Cellulose, hemicellulose and lignin composed lignocellulose with a three-dimensional complex structure (Zhao et al. 2012). Cellulose and hemicellulose are macromolecules from different saccharides. The catabolites of cellulose are cellobiose and glucose (Perez et al. 2002). Hemicelluloses are a heterogeneous group of polysaccharides and are comprised of glucose, mannose, and xylose as well as some polymers such as glucomannans and xyloglucan (Scheller and Ulvskov 2010). Resistant starch also consists of glucose. Therefore, degradation products: glucose, mannose and xylose as indicators were chosen to illustrate the lignocellulolytic effects of the probiotics. The increased amount of the monosaccharides at 6 h can be explained by breakdown of cellulose and hemicellulose during the first 6 h. Microbial consumption may be a result of the decrease of these three monosaccharides. The result of HPLC suggested the inoculated probiotics synthesized a high-activity and complex carbohydrase system that synergistically works to disturb the structure of the fiber-starch-protein matrix and then breakdown lignocellulose content into corresponding monosaccharides in both CDDGS and RDDGS. Probiotics took advantage of these monosaccharides to proliferate and produce beneficial metabolites, thereby improving value-added utilization of the lignocellulosic biomass.

In summary, solid-state fermentation with the combination of probiotics effectively improved protein (CP, TCA and small-sized peptides) and reduced lignocellulose (cellulose, lignin, NDF and ADF) content at different levels in both DDGS. The number of probiotics, enzymes, and organic acids remarkably increased after fermentation. Microscopy revealed that the microstructure of the two DDGS were decomposed, thus facilitating their in vitro digestibility. SDS-PAGE and HPLC further confirmed the degradation of the fiber-starch-protein aggregates. Therefore, our results suggest that probiotic SSF provides an effective method for increasing utilization of the by-products from the ethanol and wine industry.

## Additional file

**Additional file 1: Table S1.** Biochemical tests of CW4. **Table S2.** BLAST results of CWLP.
