# Supplementary material for: Solid-state fermentation of distilled dried grain with solubles with probiotics for degrading lignocellulose and upgrading nutrient utilization
Source: AMB Express. 2018 Nov 26;8:188. doi: 10.1186/s13568-018-0715-z (PMC6261088; doi:10.1186/s13568-018-0715-z)
Supplement: Supplementary file 1 — Additional file 1: Table S1. Biochemical tests of CW4. Table S2. BLAST results of CWLP. [file 13568_2018_715_MOESM1_ESM.docx]

**AMB Express**

**Solid-state fermentation of** **distilled dried grain with solubles with probiotics for degrading lignocellulose and upgrading nutrient utilization**

**Cheng Wang^1, 5^ • Weifa Su^1, 5^ • Yu Zhang^1, 5^ • Lihong Hao^1, 5^ • Fengqin Wang^1, 5^ • Zeqing Lu****^1,5^ • Jian Zhao^2, 5^ • Xuelian Liu^3, 5^ • Yizhen Wang^1, 6^**

^1^National Engineering Laboratory of Biological Feed Safety and Pollution Prevention and Control, Key Laboratory of Animal Nutrition and Feed, Ministry of Agriculture, Key Laboratory of Animal Nutrition and Feed Science of Zhejiang Province, Institute of Feed Science, Zhejiang University, 866 Yuhang Tang Road, Hangzhou Zhejiang 310058, P.R. China

^2^Ningbo Academy of Agricultural Science, 19 Houde Road, Ningbo 315000, China

^3^Beijing Dabeinong Technology Group Co., Ltd.

^5^ Cheng Wang: [553150821@qq.com](mailto:553150821@qq.com); Weifa Su: [508364867@qq.com](mailto:508364867@qq.com); Yu Zhang: 494941469qq.com; Lihong Hao: 1606658944qq.com; Fenqin Wang: [wangfq@zju.edu.cn](mailto:wangfq@zju.edu.cn); Zeqinq Lu: [zqlu2012@zju.edu.cn](mailto:zqlu2012@zju.edu.cn); Jian Zhao: [akjw2002@163.com](mailto:akjw2002@163.com); Xuelian Liu: linxuelian@dbn.com.cn ^6^Corresponding author: [yzwang321@zju.edu.cn](mailto:yzwang321@zju.edu.cn)

**CW4**

**CGMCC No:12825**

Morphological, biochemical tests and sequence analysis showed that the identified strain was ***Bacillus subtilis.***

Table S1: biochemical tests of CW4

| Item | [Identification result](http://dict.cnki.net/dict_result.aspx?searchword=%e9%89%b4%e5%ae%9a%e7%bb%93%e6%9e%9c&tjType=sentence&style=&t=identification+result) |
| --- | --- |
| Contact enzyme | + |
| Anaerobic growth | - |
| VP test | + |
| Sugar fermentation (Glucose, xylose, [arabinose](https://www.baidu.com/link?url=arh-Su-CdT6arZ9OgXtUjTBknZeMsieHpeVSKjyhxL6qYsD3wDNCkLIBoT6c7NmZ-RMOUDsmNKr_1sB0O953vmMQn3C1m5NMRTsrnukFJ67&wd=&eqid=f88c2bb600007beb0000000458d8c581), mannitol) | + |
| Gas production by glucose | - |
| Nitrate reduction | + |
| 6.5% Nacl growth | + |
| Starch hydrolysis | + |
| Gelatin liquefaction | + |

“+”: positive; “-”: negative

**CWLP**

**CGMCC No: 1.510**

16S rRNA sequence analysis showed that the identified strain was a ***Lactobacillus plantarum* strain.**

**16S rRNA: 1344bp**

TGCATCTTGATTTACATTTGAGTGAGTGGCGAACTGGTGAGTAACACGTGGGAAACCTGCCCAGAAGCGGGGGATAACACCTGGAAACAGATGCTAATACCGCATAACAACTTGGACCGCATGGTCCGAGCTTGAAAGATGGCTTCGGCTATCACTTTTGGATGGTCCCGCGGCGTATTAGCTAGATGGTGGGGTAACGGCTCACCATGGCAATGATACGTAGCCGACCTGAGAGGGTAATCGGCCACATTGGGACTGAGACACGGCCCAAACTAATACGGGAGGCAGCAGTAGGGAATCTTCCACAATGGACGAAAGTCTGATGGAGCAACGCCGCGTGAGTGAAGAAGGGTTTCGGCTCGTAAAACTCTGTTGTTAAAGAAGAACATATCTGAGAGTAACTGTTCAGGTATTGACGGTATTTAACCAGAAAGCCACGGCTAACTACGTGCCAGCAGCCGCGGTAATACGTAGGTGGCAAGCGTTGTCCGGATTTATTGGGCGTAAAGCGAGCGCAGGCGGTTTTTTAAGTCTGATGTGAAAGCCTTCGGCTCAACCGAAGAAGTGCATCGGAAACTGGGAAACTTGAGTGCAGAAGAGGACAGTGGAACTCCATGTGTAGCGGTGAAATGCGTAGATATATGGAAGAACACCAGTGGCGAAGGCGGCTGTCTGGTCTGTAACTGACGCTGAGGCTCGAAAGTATGGGTAGCAAACAGGATTAGATACCCTGGTAGTCCATACCGTAAACGATGAATGCTAAGTGTTGGAGGGTTTCCGCCCTTCAGTGCTGCAGCTAACGCATTAAGCATTCCGCCTGGGGAGTACGGCCGCAAGGCTGAAACTCAAAGGAATTGACGGGGGCCCGCACAAGCGGTGGAGCATGTGGTTTAATTCGAAGCTACGCGAAGAACCTTACCAGGTCTTGACATACTATGCAAATCTAAGAGATTAGACGTTCCCTTCGGGGACATGGATACAGGTGGTGCATGGTTGTCGTCAGCTCGTGTCGTGAGATGTTGGGTTAAGTCCCGCAACGAGCGCAACCCTTATTATCAGTTGCCAGCATTAAGTTGGGCACTCTGGTGAGACTGCCGGTGACAAACCGGAGGAAGGTGGGGATGACGTCAAATCATCATGCCCCTTATGACCTGGGCTACACACGTGCTACAATGGATGGTACAACGAGTTGCGAACTCGCGAGAGTAAGCTAATCTCTTAAAGCCATTCTCAGTTCGGATTGTAGGCTGCAACTCGCCTACATGAAGTCGGAATCGCTAGTAATCGCGGATCAGCATGCCGCGGTGAATACGTTCCCGGGCCTTGTACACACCGCCCGTCTAC

Table S2: BLAST results of CWLP

| Description | [Max score](https://blast.ncbi.nlm.nih.gov/Blast.cgi?CMD=Get&ALIGNMENTS=10&ALIGNMENT_VIEW=Pairwise&DATABASE_SORT=0&DESCRIPTIONS=10&DYNAMIC_FORMAT=on&FIRST_QUERY_NUM=0&FORMAT_OBJECT=Alignment&FORMAT_PAGE_TARGET=&FORMAT_TYPE=HTML&GET_SEQUENCE=yes&I_THRESH=&LINE_LENGTH=60&MASK_CHAR=2&MASK_COLOR=1&NUM_OVERVIEW=10&PAGE=MegaBlast&QUERY_INDEX=0&QUERY_NUMBER=0&RESULTS_PAGE_TARGET=&RID=2YWRW7B0015&SHOW_LINKOUT=yes&SHOW_OVERVIEW=yes&STEP_NUMBER=&OLD_VIEW=false&DISPLAY_SORT=1&HSP_SORT=1) | [Total score](https://blast.ncbi.nlm.nih.gov/Blast.cgi?CMD=Get&ALIGNMENTS=10&ALIGNMENT_VIEW=Pairwise&DATABASE_SORT=0&DESCRIPTIONS=10&DYNAMIC_FORMAT=on&FIRST_QUERY_NUM=0&FORMAT_OBJECT=Alignment&FORMAT_PAGE_TARGET=&FORMAT_TYPE=HTML&GET_SEQUENCE=yes&I_THRESH=&LINE_LENGTH=60&MASK_CHAR=2&MASK_COLOR=1&NUM_OVERVIEW=10&PAGE=MegaBlast&QUERY_INDEX=0&QUERY_NUMBER=0&RESULTS_PAGE_TARGET=&RID=2YWRW7B0015&SHOW_LINKOUT=yes&SHOW_OVERVIEW=yes&STEP_NUMBER=&OLD_VIEW=false&DISPLAY_SORT=2&HSP_SORT=1) | [Query cover](https://blast.ncbi.nlm.nih.gov/Blast.cgi?CMD=Get&ALIGNMENTS=10&ALIGNMENT_VIEW=Pairwise&DATABASE_SORT=0&DESCRIPTIONS=10&DYNAMIC_FORMAT=on&FIRST_QUERY_NUM=0&FORMAT_OBJECT=Alignment&FORMAT_PAGE_TARGET=&FORMAT_TYPE=HTML&GET_SEQUENCE=yes&I_THRESH=&LINE_LENGTH=60&MASK_CHAR=2&MASK_COLOR=1&NUM_OVERVIEW=10&PAGE=MegaBlast&QUERY_INDEX=0&QUERY_NUMBER=0&RESULTS_PAGE_TARGET=&RID=2YWRW7B0015&SHOW_LINKOUT=yes&SHOW_OVERVIEW=yes&STEP_NUMBER=&OLD_VIEW=false&DISPLAY_SORT=4&HSP_SORT=0) | [E value](https://blast.ncbi.nlm.nih.gov/Blast.cgi?CMD=Get&ALIGNMENTS=10&ALIGNMENT_VIEW=Pairwise&DATABASE_SORT=0&DESCRIPTIONS=10&DYNAMIC_FORMAT=on&FIRST_QUERY_NUM=0&FORMAT_OBJECT=Alignment&FORMAT_PAGE_TARGET=&FORMAT_TYPE=HTML&GET_SEQUENCE=yes&I_THRESH=&LINE_LENGTH=60&MASK_CHAR=2&MASK_COLOR=1&NUM_OVERVIEW=10&PAGE=MegaBlast&QUERY_INDEX=0&QUERY_NUMBER=0&RESULTS_PAGE_TARGET=&RID=2YWRW7B0015&SHOW_LINKOUT=yes&SHOW_OVERVIEW=yes&STEP_NUMBER=&OLD_VIEW=false&DISPLAY_SORT=0&HSP_SORT=0) | [Ident](https://blast.ncbi.nlm.nih.gov/Blast.cgi?CMD=Get&ALIGNMENTS=10&ALIGNMENT_VIEW=Pairwise&DATABASE_SORT=0&DESCRIPTIONS=10&DYNAMIC_FORMAT=on&FIRST_QUERY_NUM=0&FORMAT_OBJECT=Alignment&FORMAT_PAGE_TARGET=&FORMAT_TYPE=HTML&GET_SEQUENCE=yes&I_THRESH=&LINE_LENGTH=60&MASK_CHAR=2&MASK_COLOR=1&NUM_OVERVIEW=10&PAGE=MegaBlast&QUERY_INDEX=0&QUERY_NUMBER=0&RESULTS_PAGE_TARGET=&RID=2YWRW7B0015&SHOW_LINKOUT=yes&SHOW_OVERVIEW=yes&STEP_NUMBER=&DISPLAY_SORT=3&HSP_SORT=3) | Accession |
| --- | --- | --- | --- | --- | --- | --- |
| [*Lactobacillus plantarum* strain UNIFG122 16S ribosomal RNA gene, partial sequence](https://blast.ncbi.nlm.nih.gov/Blast.cgi#alnHdr_807781557) | 2466 | 2466 | 99% | 0.0 | 99% | [KP899091.1](https://www.ncbi.nlm.nih.gov/nucleotide/KP899091.1?report=genbank&log$=nucltop&blast_rank=1&RID=2YWRW7B0015) |
| [*Lactobacillus plantarum* strain UNIFG108 16S ribosomal RNA gene, partial sequence](https://blast.ncbi.nlm.nih.gov/Blast.cgi#alnHdr_807781550) | 2466 | 2466 | 99% | 0.0 | 99% | [KP899084.1](https://www.ncbi.nlm.nih.gov/nucleotide/KP899084.1?report=genbank&log$=nucltop&blast_rank=2&RID=2YWRW7B0015) |
| [*Lactobacillus plantarum* strain UNIFG107 16S ribosomal RNA gene, partial sequence](https://blast.ncbi.nlm.nih.gov/Blast.cgi#alnHdr_807781549) | 2466 | 2466 | 99% | 0.0 | 99% | [KP899083.1](https://www.ncbi.nlm.nih.gov/nucleotide/KP899083.1?report=genbank&log$=nucltop&blast_rank=3&RID=2YWRW7B0015) |
| [*Lactobacillus plantarum* strain HL14403 16S ribosomal RNA gene, partial sequence](https://blast.ncbi.nlm.nih.gov/Blast.cgi#alnHdr_1280037503) | 2460 | 2460 | 99% | 0.0 | 99% | [KY417131.1](https://www.ncbi.nlm.nih.gov/nucleotide/KY417131.1?report=genbank&log$=nucltop&blast_rank=4&RID=2YWRW7B0015) |
| [*Lactobacillus plantarum* strain 3B2 16S ribosomal RNA gene, partial sequence](https://blast.ncbi.nlm.nih.gov/Blast.cgi#alnHdr_1279653780) | 2460 | 2460 | 99% | 0.0 | 99% | [MG561858.1](https://www.ncbi.nlm.nih.gov/nucleotide/MG561858.1?report=genbank&log$=nucltop&blast_rank=5&RID=2YWRW7B0015) |
| [*Lactobacillus plantarum* strain NWAFU1529 16S ribosomal RNA gene, partial sequence](https://blast.ncbi.nlm.nih.gov/Blast.cgi#alnHdr_1279336855) | 2460 | 2460 | 99% | 0.0 | 99% | [MG551199.1](https://www.ncbi.nlm.nih.gov/nucleotide/MG551199.1?report=genbank&log$=nucltop&blast_rank=6&RID=2YWRW7B0015) |
| [*Lactobacillus plantarum* strain HY-08 16S ribosomal RNA gene, partial sequence](https://blast.ncbi.nlm.nih.gov/Blast.cgi#alnHdr_1278992657) | 2460 | 2460 | 99% | 0.0 | 99% | [MG547899.1](https://www.ncbi.nlm.nih.gov/nucleotide/MG547899.1?report=genbank&log$=nucltop&blast_rank=7&RID=2YWRW7B0015) |
| [*Lactobacillus plantarum* strain NWAFU1285 16S ribosomal RNA gene, partial sequence](https://blast.ncbi.nlm.nih.gov/Blast.cgi#alnHdr_1276390682) | 2460 | 2460 | 99% | 0.0 | 99% | [MG462194.1](https://www.ncbi.nlm.nih.gov/nucleotide/MG462194.1?report=genbank&log$=nucltop&blast_rank=8&RID=2YWRW7B0015) |
| [*Lactobacillus plantarum* strain NWAFU1227 16S ribosomal RNA gene, partial sequence](https://blast.ncbi.nlm.nih.gov/Blast.cgi#alnHdr_1276390610) | 2460 | 2460 | 99% | 0.0 | 99% | [MG462122.1](https://www.ncbi.nlm.nih.gov/nucleotide/MG462122.1?report=genbank&log$=nucltop&blast_rank=9&RID=2YWRW7B0015) |
| [*Lactobacillus plantarum* strain NWAFU1203 16S ribosomal RNA gene, partial sequence](https://blast.ncbi.nlm.nih.gov/Blast.cgi#alnHdr_1276390586) | 2460 | 2460 | 99% | 0.0 | 99% | [MG462098.1](https://www.ncbi.nlm.nih.gov/nucleotide/MG462098.1?report=genbank&log$=nucltop&blast_rank=10&RID=2YWRW7B0015) |
